# Development and Optimization of an RNA-Isolating Protocol for Mammalian Spermatozoa

**DOI:** 10.3390/ijms262211171

**Published:** 2025-11-19

**Authors:** El Oulidi Mounia, Azzouzi Naoual, Ravel Celia, Hassani Idrissi Hind, Habbane Mouna, Fieni Francis, Galibert Francis, Akhouayri Omar

**Affiliations:** 1 Laboratory of Biology and Health, Ibn Tofail University, Kenitra 14000, Morocco; mounia.eloulidi@uit.ac.ma (E.O.M.); hind.hassaniidrissi@uit.ac.ma (H.I.H.); mouna.habbane@uit.ac.ma (H.M.); omar.akhouayri@uit.ac.ma (A.O.); 2 Institut de Génétique et Développement de Rennes, UMR6290-CNRS, Université de Rennes, 35000 Rennes, France; naoual.azzouzi@univ-rennes1.fr; 3 CHU de RENNES-Service de Biologie de la Reproduction-CECOS, Institut National de la Santé et de la Recherche Médicale, Ecole des Hautes Etudes en Santé Publique, Institut de Recherche en Santé, Environnement et Travail, Université de Rennes, UMR_S1085, 35000 Rennes, France; celia.ravel@chu-rennes.fr; 4 Veterinary Reproduction Service ONIRIS of Nantes, 44300 Nantes, France

**Keywords:** sperm, RNA isolation, mammals, NucleoSpin RNA II kit, dithiothreitol, TRIzol

## Abstract

The RNAs present in spermatozoa play a crucial role in reproduction and embryonic development. They represent a promising diagnostic tool for assessing male infertility. However, their extraction is challenging due to their low concentration and highly condensed chromatin structure, as well as the presence of numerous cellular contaminants. These challenges vary across species and require the development of an optimized and reliable isolation method to obtain high-quality RNAs, which is essential for further molecular analyses regarding the roles played by these RNAs. This study evaluated two RNA extraction methods for spermatozoa in humans and other mammals (dogs, stallions, and bulls): a standard method using the NucleoSpin RNA^®^ II kit (Macherey-Nagel) and an optimized method that combined this kit with dithiothreitol and TRIzol™ pretreatment. In addition, the samples underwent pre-purification to eliminate somatic cells. The optimized method produced a significantly higher total RNA yield along with better purity, which was confirmed by the absence of the 18S and 28S ribosomal RNA peaks, indicating the absence of somatic cell contamination. Additionally, RT-PCR was performed to validate the presence of sperm-specific markers. The quality of the extracted RNAs was assessed by sequencing the mRNA encoding the human olfactory receptor OR1D2 and observing the resulting band on an agarose slab gel with a size corresponding to its entire open reading frame. By addressing long-standing challenges in sperm RNA isolation, our method provides an easy and standardized technique for research in reproductive biology and for advancing our understanding of paternal contributions to transgenerational inheritance.

## 1. Introduction

Spermatogenesis is a highly regulated developmental process comprising three major developmental stages that are well-conserved in mammals. The first stage is a phase of multiplication and division via mitosis of spermatogonia. The second stage involves meiotic division of spermatocytes, resulting in haploid round spermatids. Finally, the last stage is spermiogenesis, where elongated spermatids undergo major morphological changes to produce mature spermatozoa [1]. During this differentiation into spermatozoa, the cell’s transcriptional activity stops. Although the condensed nucleus of spermatozoa is thought to be in a transcriptionally inert state, sperm RNAs have been identified in mammalian species, including human. These RNAs may influence embryo quality and viability and, therefore, may impact the outcomes of assisted reproductive techniques [2].

While RNA synthesis takes place at the beginning, it ceases completely with the compaction of DNA, a process that occurs to ensure genetic stability [3,4,5,6]. The presence of mRNAs in human spermatozoa, such as those coding for olfactory receptors, has been documented [7]. In addition, the presence of all sorts of non-coding RNAs in human spermatozoa is now well established, and these RNAs are known to be involved in epigenetic inheritance. Long non-coding RNAs (>200 nucleotides) are responsible for maintaining epigenetic memory through several mechanisms, including the regulation of DNA methylation, chromatin remodeling, and histone modifications. Small non-coding RNAs (<200 nucleotides) include microRNAs (miRNAs), piwi-interacting RNAs (piRNAs), and endogenous small interfering RNAs (endo-siRNAs), as well as other types of small non-coding RNAs such as tRNAs, degraded rRNAs, and small nucleolar RNAs [8,9,10]. In human and other mammalian sperm cells, RNAs are primarily localized in the sperm nucleus and in the midpiece, a region rich in mitochondria essential for sperm motility [11,12,13,14].

Numerous studies have clearly demonstrated that sperm RNAs are not mere transcriptional remnants of spermatogenesis; rather, they play essential roles in male fertility, fertilization, early embryonic development, and epigenetic inheritance [15,16,17,18].

Various techniques have been used to analyze these RNAs, such as RT-PCR, microarrays, and RNA sequencing. These analyses are crucial in fundamental research and reproductive medicine. However, their success depends on obtaining a large quantity of RNAs devoid of contaminants such as salts, proteins, DNA, and organic solvents. Moreover, these RNAs must be in their native structure, i.e., not degraded by the extraction process itself.

The extraction of RNAs from human and mammalian sperm cells presents several challenges due to the unique biological characteristics of these cells. Semen is a heterogeneous mixture that contains not only spermatozoa but also somatic cells (e.g., leukocytes, residual germ cells, and epithelial cells), which contain approximately 200 times more RNAs than spermatozoa [19], with an estimate of 10 to 100 fg per cell. Moreover, these RNAs, which include ribosomal RNAs, appear to be highly degraded by intrinsic ribonucleases [20,21,22]. Therefore, a rigorous purification step to isolate spermatozoa that are free of contaminants is essential.

Another major obstacle lies in the strong condensation of nuclear DNA due to the replacement of histones by disulfide-rich protamines. This process ensures DNA compaction and limits the accessibility to RNA molecules, making their extraction even more complex [6]. Finally, sperm RNAs exhibit significant heterogeneity, including a number of small non-coding RNAs (such as miRNAs and piRNAs), mRNAs, and rRNA fragments, raising question regarding the persistence of some intact 18S and 28S molecules that are undetected. tRNA biogenesis occurs during post-testicular sperm maturation and can regulate the expression of transcripts driven by endogenous retroelements that modify the sperm epigenome in mammals [23].

The biological and physiological differences between the spermatozoa of different mammalian species add further complexity to RNA extraction. These differences include the size, structure, and chromatin compaction levels of sperm, which vary considerably among species [24]. Consequently, most studies have developed and adjusted species-specific protocols, including those for humans [25], bulls [26], stallions [27], rats [28], pigs [29], goats [30], and bovines [31].

Currently, there is no commercially available standardized method specifically designed for the extraction of spermatozoal RNAs, which explains the high variability in yields obtained with existing protocols. Consequently, researchers have developed their own protocol tailored to each species, making the generalized application of existing methods particularly complex. Overcoming these obstacles requires the development of an optimized and robust protocol that is capable of maximizing the recovery of high-quality RNAs (as defined above) regardless of the species studied. Furthermore, the implementation of rigorous quality controls is essential to ensure that the extracted RNAs are suitable for sensitive molecular analyses aimed at analyzing their roles.

In this context, we have developed a standardized and robust method that is specifically optimized for spermatic RNA extraction. Our approach aims to reduce inter-experimental variability, improve the reproducibility of results, and facilitate the study of spermatozoal RNAs, both in fundamental research and in clinical settings.

## 2. Results

The aim of this study was to develop a reliable RNA extraction method which is applicable to different mammalian species. To this end, we compared the efficiency of two distinct approaches in the extraction of RNAs from human and other mammalian spermatozoa (dogs, stallions, and bulls): a standard protocol based on the NucleoSpin RNA II kit (Macherey-Nagel) and an optimized protocol that included the addition of dithiothreitol (DTT) to the RA1 lysis buffer, along with an additional pretreatment step using the TRIzol reagent. Moreover, to ensure the absence of somatic cell contamination, all the semen samples were first purified using a density gradient.

**Figure 1:**
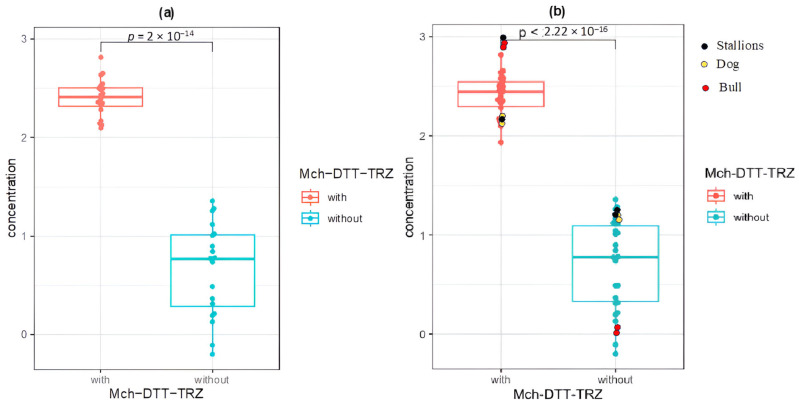
Comparison of RNA concentrations extracted from the spermatozoa of human and other mammals using the combined Macherey-Nagel + DTT + TRIzol method (Mch–DTT–TRZ labeled “with”) versus the standard kit alone (labeled “without”). Panel (**a**) shows results from human spermatozoa, and panel (**b**) shows results from all species analyzed (humans, bulls, stallions, and dogs). Different colors indicate the species analyzed.

Table 1 shows the concentrations and absorbance ratios of the total RNAs extracted from the different samples, and Figure 1 shows their statistical treatment. Significant differences were observed in favor of the combined extraction method we developed. Interestingly, the concentrations of the different samples showed a more homogeneous distribution with our method.

The statistical analyses indicated a highly significant difference between the two compared methods, with a highly significant *p*-value of 10^−8^ for the raw human sperm RNA concentrations. For a clearer and more intuitive representation, the raw values were transformed into base 10 logarithms, yielding an even more significant *p*-value of 2 × 10^−14^ (Figure 1, panel a). Panel b displays the RNA concentration values for all the studied mammals. The observed difference remained highly significant, with a *p*-value of 2.22 × 10^−16^. These results demonstrate that the optimized NucleoSpin RNA II + DTT + TRIzol method was significantly more effective for sperm RNA extraction from both humans and other mammals (Figure 1, panel b).

The box plots illustrate the RNA concentrations obtained using the combined Mch(Macherey-Nagel)-DTT-TRZ method (labeled “with”) relative to the method with only the kit (labeled “without”). Panel (a) displays the results for humans, while panel (b) presents the results for the entire set of mammals (humans, bulls, stallions, and dogs). Dots with different colors are used to distinguish the species.

The *Y*-axis represents RNA concentrations and the horizontal bars indicate the medians. Welch’s t-test revealed highly significant *p*-values: *p* = 2 × 10^−14^ for human and *p* = 2.22 × 10^−16^ for all mammals (humans, bulls, stallions, and dogs).

We also performed an analysis of variance (ANOVA) and obtained a *p*-value of 10^−5^ and variance of 30.50 for human only and a *p*-value of 10^−5^ and variance of 37.24 for all mammals.

**Figure 2:**
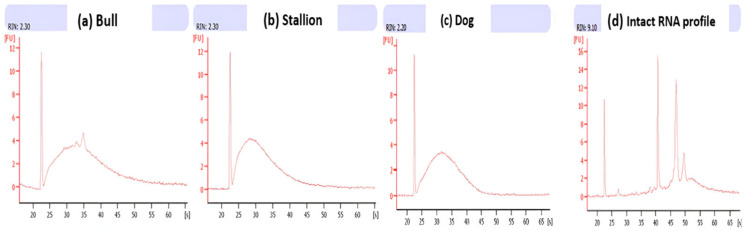
Profiles of RNA samples extracted using our method and analyzed using the BioAnalyzer 2100. Profiles for (**a**) bulls, (**b**) stallions, (**c**) dogs, and (**d**) typical profile obtained with somatic cells. (RIN: RNA Integrity Number).

Not surprisingly, the profiles obtained with the BioAnalyzer 2100 (Agilent, Santa Clara, CA, USA) did not show the usual two peaks for ribosomal RNAs (18S and 28S), indicating the absence of somatic cell contamination (Figure 2) as well as the absence of intact or detectable ribosomal RNAs [27].

**Figure 3:**
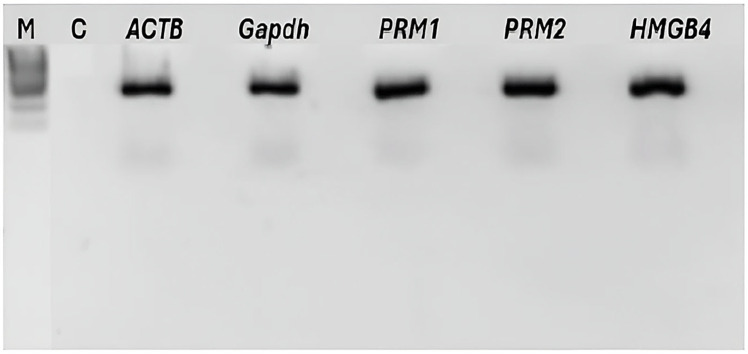
RT-PCR analysis of the *ACTB* (beta-actin), *GAPDH* (glyceraldehyde-3-phosphate dehydrogenase), *PRM1* (protamine 1), *PRM2* (protamine 2), and *HMGB4* (high-mobility group protein B4) genes from a human sperm sample. The RNAs were extracted using the Macherey-Nagel kit, DTT, and TRIzol following our method. Lane M, size markers; lane C, negative control.

All the RT-PCR products corresponding to sperm-specific genes—namely, *PRM1* (protamine 1), *PRM2* (protamine 2), and *HMGB4* (high-mobility group protein B4)—were analyzed using gel electrophoresis. As shown in Figure 3, a clear and intense band was observed for each of these genes and for *ACTB* (beta-actin) and *GAPDH* (glyceraldehyde-3-phosphate dehydrogenase), which were used as the positive controls.

**Figure 4:**
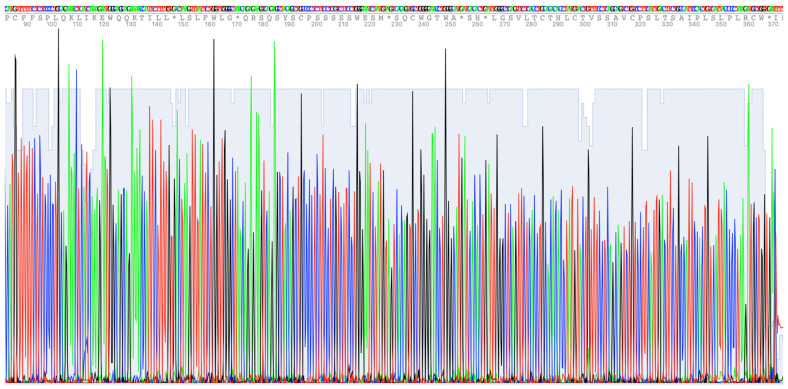
Part of the profile obtained from the sequence of the human olfactory receptor *OR1D2/hOR17-4*. * This means stop codon.

The quality of the extracts was also checked by sequencing the mRNA of the human olfactory receptor *OR1D2* (also named hOR17-4) (Figure 4), whose cognate protein is likely essential for egg fertilization [32].

The absence of a background allows for base calling without ambiguity, and this absence of ambiguity in the identification of the bases demonstrates the quality of the extraction method used throughout the preparation, from the collection of the semen samples to the sequencing.

**Figure 5:**
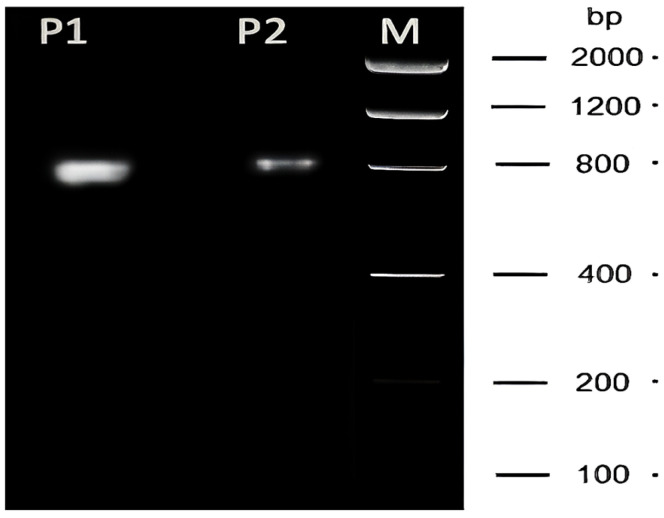
Slab gel electrophoresis showing an RT-PCR product whose size nearly covers the complete open reading frame of *OR1D2*/*hOR17-4* (a human olfactory receptor) from human sperm samples (P1 and P2). The figure also shows the absence of background signal. Lane M represents the size markers.

The quality of the extracted RNAs was further demonstrated by the size of the RT-PCR product (about 800 nucleotides), which nearly covers the entire coding sequence of the olfactory receptor *OR1D2*, as shown in Figure 5.

While most authors would insist on the typical absence of intact rRNAs in spermatozoa, in which only small RNAs (such as miRNAs and piRNAs) subsist, we believe that an appropriate method such as the one we developed in this work might offer a new perspective. The size of the RT-PCR product shown in Figure 5 provides the ultimate proof of the quality of the extraction method we developed, which can provide new possibilities for research.

## 3. Discussion

In this paper, we report the development of a novel approach that can extract large quantities of RNAs with high quality from the semen samples of different mammals, including humans. The extraction of RNAs from spermatozoa represents a unique challenge due to the specific biological characteristics of these cells. Unlike somatic cells, spermatozoa contain an extremely low amount of RNAs, mixed with a high proportion of genomic DNA. Moreover, sperm chromatin is highly condensed and stabilized by protamines, which significantly limits access to nucleic acids [33]. This challenge is further accentuated by the presence of a variety of diploid cells in the ejaculate, making the specific isolation of RNAs from spermatozoa particularly difficult [25,34]. Moreover, these challenges vary across different species. These characteristics highlight the need to develop an optimized and adapted extraction method to obtain a large quantity of RNAs with the highest possible quality in order to perform reliable subsequent molecular and functional analyses regarding their roles, if any.

Several studies have shown that the removal of somatic cells prior to extraction is an essential step to improve the purity of the recovered RNAs. For human samples, a preliminary step including centrifugation in a density gradient with the use of commercial kits, such as Qiagen, Macherey-Nagel, and Life Technologies, has yielded satisfactory results [3,7,28,35,36].

However, these kits have proven insufficient for many animal species due to the strong chromatin condensation. Faced with this constraint, the use of TRIzol™ has been recommended to improve cell lysis and RNA extraction in some species, such as in pigs [32], dogs [37], or stallions and bulls [38].

In contrast, some studies have observed that spermatozoa show resistance to guanidinium thiocyanate (GITC)-based reagents, such as TRIzol and the lysis buffers of commercial kits leads to low RNA yields; this has been noted for various species, including humans [28], goats [30], cattle [39], and chickens [40]. To overcome these obstacles, specific adjustments to the extraction protocols have been proposed, including the addition of reducing agents such as *β*-mercaptoethanol or DTT. These agents break disulfide bonds in proteins and improve the lysis of sperm heads, where the majority of RNAs are located [25,30,31,39]. However, the efficacy of these reducing agents strongly depends on the pH of the medium. In this context, tris(2-carboxyethyl) phosphine has proven particularly effective as an GITC-based reagent at an acidic pH [41,42].

Besides chemical treatments, mechanical methods have been used to enhance sperm lysis. Examples of these methods include passage through a needle and syringe in cattle [42] and the use of stainless-steel beads in rats [28,41], which mechanically alters the compact structure of spermatozoa and facilitates RNA release. Finally, some researchers have suggested combining TRIzol extraction with column purification, such as the RNeasy Mini Kit (Qiagen, Hilden, Germany) [27,33].

The improvements achieved by our method resulted from the contribution of several essential elements: the addition of DTT to the RA1 lysis buffer of the NucleoSpin RNA II kit, the addition of guanidinium thiocyanate, and the adjustment to an optimal pH. As a reducing agent, DTT breaks disulfide bonds, facilitating the complete lysis of sperm heads where RNAs are primarily located. Simultaneously, DTT inhibits ribonuclease activity [43].

Moreover, the TRIzol™ treatment, with its chaotropic properties, improves cell lysis and allows for the efficient separation of RNAs in the aqueous phase. Lastly, the final purification step using silica membrane columns provided by the NucleoSpin RNA II kit removes residual contaminants (DNA, proteins, salts, and inorganic solvents), ensuring high-purity RNAs.

Table 1 and Figure 1 provide the RNA concentrations and absorbance ratios (OD260/280 and OD260/230) in detail, along with a comparison of the yields obtained using our optimized method to those obtained using the standard protocol. The data correspond to the RNAs extracted from the samples of twenty humans, two dogs, two stallions, and two bulls. Together, these data clearly demonstrate the superiority of our approach, which produced higher and more homogeneous RNA yields.

Even more important than the quality of the proposed method are the sequencing results for the mRNA encoding the human olfactory receptor *OR1D2*. In addition to being expressed in the nose, as all other ORs, OR1D2 is one of the subsets of ORs expressed in sperm cells. Of interest, OR1D2 has been shown to be involved in sperm chemotaxis [44]. Of note, according to the results obtained by Spehr [44], it is the olfactory receptor protein that is important, not its mRNA.

Numerous studies have clearly demonstrated that sperm RNAs are not mere transcriptional remnants of spermatogenesis, but play essential roles in male fertility, egg fertilization, early embryonic development, and epigenetic inheritance [10,15,16,17,19]. At this point, we can ask a rather iconoclast question regarding the absence of intact ribosomal RNAs and protein synthesis: is it possible that some intact rRNA molecules do exist undetected (Figure 2) because of the large presence of fragmented molecules? To date, no protein synthesis has been observed in sperm cells. Could we suggest that intact messenger RNAs (such as the mRNA encoding OR1D2 (Figure 4)) and/or intact ribosomal RNA molecules constitute the wake-up signal for the still-dormant cellular machinery of a just-fertilized egg, and that fertilization will abort in their absence? It is with these interrogations in mind that we thought it would be necessary to develop a suitable and reliable extraction method for sperm cell RNAs.

To the best of our knowledge, this is the first study to assess the effectiveness of the NucleoSpin RNA II kit (Macherey-Nagel) for the extraction of sperm RNAs. Although originally designed for conventional tissues, this kit has proven to be well suited for human spermatozoa, a highly compacted and RNA-poor cell type.

However, the true significance of this optimized protocol lies in its applicability to the study of spermatozoa from other mammals such as bulls, dogs, and stallions, suggesting its broader implications in the field of reproductive biology.

Our protocol, validated across phylogenetically diverse mammals, can pave the way for large-scale, comparative inter-species studies to unravel the conserved and divergent roles of sperm RNAs in fertilization and evolution.

Furthermore, by ensuring high RNA purity and yield while eliminating somatic cell contamination, this method unlocks the potential for highly sensitive downstream applications. It may enable more reliable biomarker discovery for male fertility diagnostics, allow for sophisticated epigenetic profiling to understand paternal contributions to transgenerational inheritance, and facilitate advanced techniques like single-cell RNA sequencing. By providing a reliable tool to access the sperm transcriptome, we anticipate this protocol will accelerate discovery, promote future studies on the functional roles of sperm RNAs, and ultimately expand the frontiers of reproductive genomics.

## 4. Materials and Methods

### 4.1. Sampling

The samples were provided by the GERMETHEQUE Biobank, which is dedicated to human fertility. Human semen samples (*n* = 20) were obtained from men who consulted the CECOS laboratory (Center for the Study and Conservation of Human Eggs and Sperm) in Rennes. All the samples had normal spermograms (sperm count, motility, vitality, and morphology analyses were performed according to the 2021 World Health Organization (WHO) guidelines). For all samples, the parameters were presented with the semen volume (>1.4 mL), total sperm number (>39 × 10^6^ per ejaculate), total motility (>42%) associated with progressive motility (>30%), vitality (>54%), and normal forms (>4%). The participants were informed about the nature of the study and provided informed consent for the use of the residual portion of their samples after the spermogram and spermocytogram analyses. Ethical approval (CP-GM n°20151117) was obtained for this study. Additionally, semen samples were collected from two bulls, two stallions, and two dogs at Nantes Veterinary School’s reproduction service.

**Figure 6:**
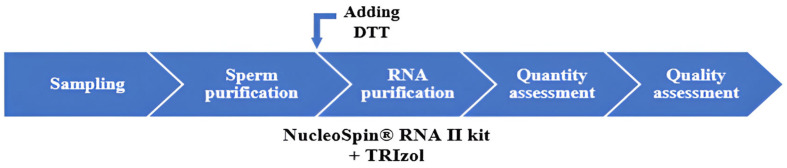
Schematic view of the whole protocol.

A schematic overview summarizing the workflow of the entire protocol is shown in Figure 6.

### 4.2. Sperm Cell Purification via Density Gradient

Purification was performed using the density gradient method, with a PureSperm^®^ kit (Nidacon International AB, Gothenburg, Sweden) for the human samples and BoviPure^®^ (Nidacon International AB, Mölndal, Sweden) for the samples from other animal species. This method involves overloading the samples on top of two solutions of different densities in a test tube. During centrifugation (1300 rpm for 20 min), the spermatozoa migrate through the gradient based on their motility and density, which are higher than those of somatic cells. They settle at the bottom of the tube and form a pellet.

All the solutions were stored at room temperature before use. The semen samples underwent liquefaction at 37 °C for at least 30 min. In a 15 mL conical tube, 1 mL of a 90% density gradient solution was added, followed by 1 mL of a 40% density gradient solution. Then, 1 mL of semen (maximum of 1.5 mL per tube) was layered on top of these solutions. To avoid disturbing the layers, centrifugation was stopped without using the brake. The upper phases were aspirated with a micropipette, leaving approximately 500 µL that contained the spermatozoa at the bottom of the tube. The pellet was transferred to a new tube, washed with 4 mL of FertiCult™ Sperm (FertiPro N.V., Beernem, Belgium), and centrifuged at 1500 rpm for 10 min. The supernatant was discarded, and 1 mL of FertiCult™ Sperm was added. The sperm concentration, which was between 0.5 and 50 million spermatozoa per mL, was determined using a hemocytometer and a Malassez cell (https://www.who.int/publications/i/item/9789240030787 (accessed on 10 October 2024)).

### 4.3. Sperm RNA Purification

To quantitatively and qualitatively improve RNA extraction, two key modifications were made to the NucleoSpin^®^ RNA II kit (Macherey-Nagel GmbH & Co. KG, Düren, Germany) protocol:

1. DTT (100 mM final concentration) was added to the RA1 lysis buffer to reduce protamine disulfide bonds. This promoted nuclear decompaction and facilitated the release of RNAs.
2. Prior to silica column purification, the samples were incubated with the TRIzol™ reagent (Invitrogen, Thermo Fisher Scientific, Waltham, MA, USA) to enhance membrane lysis, inhibit RNases, and stabilize the RNAs during extraction.

### 4.4. RNA Purification Protocol

- **Pre-lysis of Sperm Cells:** Each sample was centrifuged at 13,000 rpm for 5 min, and then 350 µL of RA1 buffer from the NucleoSpin RNA II kit (Macherey-Nagel GmbH & Co. KG, Düren, Germany) and 70 µL of DTT (100 mM) were added to the pellet. The mixture was incubated and vortexed at room temperature for 4 min.
- **TRIzol/Chloroform Treatment:** A total of 400 µL of the TRIzol reagent was added to each biological sample; the mixture was then shaken with a vortex for 10 min at room temperature. Next, 300 µL of chloroform (Sigma-Aldrich, St. Louis, MO, USA) was added and the sample was vortexed for 15 to 30 s. The sample was then centrifuged at 13,000 rpm for 4 min to separate the aqueous and organic phases.
- **Nucleic Acid Precipitation:** The aqueous phase was transferred to a sterile 1.5 mL tube, and 350 µL of 70% ethanol was added to precipitate nucleic acids, followed by mixing briefly with a vortex.
- **Column Binding:** A total of 600 µL of the precipitated sample was loaded onto a purification column (blue) placed in a collection tube from the NucleoSpin RNA II kit. This was centrifuged at 8000 rpm for 30 s to allow for nucleic acid adsorption onto the column membrane. The lysate was then discarded, and this step was repeated for the remaining sample volume.
- **Membrane Desalting:** A total of 350 µL of MDB desalting solution from the NucleoSpin RNA II kit was added to the column. Then, the mixture was centrifuged at 11,000 rpm for 1 min to remove saline impurities.
- **DNase Treatment:** The DNase mixture was prepared by adding 10 µL of reconstituted DNase I to 90 µL of DNase buffer. Next, 95 µL of this DNase mixture was added to the purification column to degrade the residual genomic DNA, and the mixture was incubated for 15 min at room temperature.
- **Washing Steps:** A total of 200 µL of RA2 wash solution from the NucleoSpin RNA II kit was added to the sample, and the mixture was centrifuged at 8000 rpm for 1 min. The filtrate was then discarded and the column was replaced in a new collection tube. Next, 600 µL of RA3 wash solution from the kit was added, the sample was centrifuged at 8000 rpm for 1 min, the filtrate was discarded, and the column was replaced in the collection tube. The washing step was repeated by adding 500 µL of RA3 and centrifuging at 8000 rpm for 1 min. The filtrate was then discarded and the column was replaced in a new collection tube.
- **Drying step:** The tube was centrifuged for 2 min at 13,000 rpm to completely dry the column membrane.
- **Elution:** During the drying step, the DNase/RNase-free water of the NucleoSpin RNA II kit was heated in a water bath at 55 °C for approximately 5 min to enhance the RNA elution efficiency. Next, 40 to 60 µL of DNase/RNase-free water was added to the center of the column for RNA elution. The sample was incubated at room temperature for 2 min and then centrifuged for 1 min at 11,000 rpm to increase the RNA yield. The RNA solution was stored at −80 °C until use.

### 4.5. Evaluation of Sperm RNA Quantity and Quality

After RNA extraction, a NanoDrop™ Spectrophotometer (Thermo Fisher Scientific, Waltham, MA, USA) was used to measure the RNA concentration in nanograms per microliter (ng/μL) and to assess its purity based on the absorbance ratios at 260/280 nm and 260/230 nm in order to detect potential contamination by proteins, salts, or organic solvents. Each measurement was performed with 2 μL of an RNA sample.

The purity and quality of the sperm RNAs were then evaluated on a Bioanalyzer 2100 (Agilent Technologies, Santa Clara, CA, USA) with an RNA nano chip (Agilent RNA 6000 Nano Kit), which generates an electrophoretic profile and calculates the RNA integrity number (RIN).

A number of RT-PCRs targeting relevant RNAs were performed to assess the quality and purity of the extraction. The list of the primers and amplification protocols used is given in the Supplementary Materials (Table S1, Figures S1 and S2). Finally, we sequenced the mRNA of *OR1D2*, a human olfactory receptor, using the Genetic Analyzer SeqStudio™ Flex (Applied Biosystems, Thermo Fisher Scientific, Waltham, MA, USA). The reaction sequences were performed using the BigDye™ Terminator V3.1 Cycle Sequencing Kit (Applied Biosystems, Thermo Fisher Scientific, Waltham, MA, USA) according to the manufacturer’s instructions (USER GUIDE Catalog no. 4337454), and the data were analyzed using 4Peaks software (February 2004 V1.8) and Geneious software (version R10.0.8).

#### Statistical Analysis

The data obtained from the two extraction methods were statistically analyzed using RStudio (Version 2023.03.1+446) to compare the RNA yields and sample quality. Comparisons were conducted using unpaired Student’s *t*-test (*p* ≤ 0.05).

## 5. Conclusions

In this study, we developed an optimized protocol integrating the NucleoSpin^®^ RNA II kit (Macherey-Nagel), dithiothreitol (DTT), and the TRIzol™ reagent, which enables the robust extraction of high-quality sperm RNAs with substantial yield and high purity. This method, which has been validated across phylogenetically diverse mammals, including humans (*Homo sapiens*), dogs (*Canis lupus familiaris*), stallions (*Equus caballus*), and bulls (*Bos taurus*), offers a standardized, species-agnostic solution for comparative inter-species studies and translational research in reproductive biology. Its simplicity, reproducibility, and elimination of somatic RNA contamination make it ideal for sensitive downstream applications such as RT-PCR, single-cell RNA sequencing, and epigenetic profiling. By overcoming long-standing challenges in sperm RNA isolation, this protocol can pave the way for advancements in understanding paternal contributions to fertility, transgenerational inheritance, and biomarker discovery, thereby expanding the frontiers in reproductive genomics.

## Figures and Tables

**Table 1:** Concentrations and absorbance of RNAs extracted using two different methods across different mammals. (OD: optical density).

Species	NucleoSpin RNA II Kit			NucleoSpin RNA II Kit -DTT-TRIzol		
	RNA Concentration (ng/μL)	OD 260/280	OD 260/230	RNA Concentration (ng/μL)	OD 260/280	OD 260/230
**Human** *n* = 20	5.47	1.36	1.23	213.38	1.87	2.40
	19.10	1.53	1.68	224.76	1.86	2.37
	7.00	1.49	1.40	192.88	1.85	2.38
	22.83	1.43	1.04	134.05	1.83	2.37
	10.21	1.54	1.22	229.29	1.85	2.43
	7.91	1.47	1.53	315.66	1.87	2.41
	18.13	1.41	1.43	336.17	1.87	2.40
	10.50	1.61	1.56	272.00	1.86	2.42
	13.16	1.40	1.34	655.25	1.91	2.35
	0.63	0.76	1.22	434.55	1.89	2.38
	0.78	1.05	0.00	318.57	1.85	2.42
	1.64	1.00	0.27	249.45	1.84	2.43
	1.35	1.13	0.75	450.53	1.87	2.40
	6.02	1.53	1.14	125.66	1.78	2.37
	5.86	1.46	1.80	148.06	1.79	2.29
	3.09	1.27	0.28	281.85	1.84	2.34
	2.06	7.32	0.35	352.44	1.88	2.38
	5.95	3.13	0.60	305.42	1.84	2.31
	1.57	2.35	0.13	230.17	1.87	2.28
	2.32	0.00	0.28	139.35	1.77	2.21
**Dog** *n* = 2	14.63	2.01	2.02	146.90	2.10	2.04
	12.87	1.74	1.43	85.71	1.84	2.69
**Stallion** *n* = 2	13.01	1.18	0.67	285.50	1.92	2.17
	11.01	0.33	0.28	380.50	1.91	2.24
**Bull** *n* = 2	3.09	4.05	0.01	402.70	1.90	2.26
	2.08	4.05	0.01	376.57	1.90	2.15

## Data Availability

The original contributions presented in this study are included in the article/Supplementary Material. Further inquiries can be directed to the corresponding author.

## Supplementary Materials

The following supporting information can be downloaded at https://www.mdpi.com/article/10.3390/ijms262211171/s1.
