# Supplementary material for: Development and Optimization of an RNA-Isolating Protocol for Mammalian Spermatozoa
Source: Int J Mol Sci. 2025 Nov 19;26(22):11171. doi: 10.3390/ijms262211171 (PMC12652690; doi:10.3390/ijms262211171)
Supplement: Supplementary file 1 [file ijms-26-11171-s001.zip › ijms-3807235-supplementary.pdf]

Supplementary data

Table S1: RT-PCR primers

Genes	Primer Sequences (5'-3')	Genomic size (pb)	cDNA size (pb)	Annealing Tempe, (°C)
ACTB	**F** AAGACCCCAGCACACTTAGC<br>**R** CCAGGAAGGAAGGCTGGAAG	550	90	60
HMGB4	**F** GCGATCGCCATGGGAAAAGA<br>**R** ACTCTTTTCCCTCTGCACCG	557		60
Gapdh	**F** GATTTGGTCGTATTGGGCGC<br>**R** AGTGATGGCATGGACTGTGG	517		60
PRM1	**F** CAGCCCACAGAGTTCCACC<br>**R** ATGGCTCTCCTCCGTGTCT	201	156	60
PRM2	**F** GTGACCCCCTCAACCAGAAC<br>**R** GCAGGTGACTTTTGCTCGTT	322	149	60

The RT-PCR experiments were optimized, and all primer sequences as well as the annealing temperatures are provided in the Supplementary Materials (Table S1). Total RNA (100–200 ng) was reverse-transcribed into cDNA using the iScript™ cDNA Synthesis Kit (Bio-Rad Laboratories, Inc., Hercules, CA, USA) under the following conditions: 25 °C for 5 min, 46 °C for 20 min, and 95 °C for 1 min. The PCR reactions (20 µL) were prepared with Type-IT® Master Mix (Qiagen, Hilden, Germany), primers (3 µM), and cDNA (20 ng), and amplified in a Bio-Rad thermal cycler using a touchdown (TD61-51) protocol. This included an initial denaturation at 94 °C for 5 min, followed by 30 cycles (94 °C for 15 s, annealing at the temperatures indicated in Table S1 for 30 s, extension at 72 °C for 60 s), and a final extension at 72 °C for 7 min. These conditions were chosen to ensure both specificity and reproducibility of the amplifications.

Figure S1. RT-PCR primers of *OR1D2* with their positions.

```
>OR1D2 range=chr17:3091858-3093196 5'pad=100 3'pad=100 strand=- repeatMasking=none
CTTTGAAGTAGCTGAAATAATTATATCGCATAAAAACTTTGTTATATTTT
TCACTTTCTTATTTTCAAAAATTATAAAATTGGGTGTAAGACATTCTTAA
TTCTAAGAAAATGTTGATTTTGCTTATCTTCATGTTTTTATTCAATTAAG
GACTTTTGGTAAACATTTGCTGGTGTTAATGTTAAAAGAGAGTTGGGGAA
ATGGATGGAGGCAACCAGAGTGAAGGTTCAGAGTTCCTTCTCCTGGGGAT
GTCAGAGAGTCCTGAGCAGCAGCGGATCCTGTTTTGGATGTTCCTGTCCA
TGTACCTGGTCACGGTGGTGGGAAATGTGCTCATCATCCTGGCCATCAGC
TCTGATTCCCGCCTGCACACCCCCGTGTACTTCTTCCTGGCCAACCTCTC
CTTCACTGACCTCTTCTTTGTCACCAACACAATCCCCAAGATGCTGGTGA
ACCTCCAGTCCCATAACAAAGCCATCTCCTATGCAGGGTGTCTGACACAG
CTCTACTTCCTGGTCTCCTTGGTGGCCCTGGACAACCTCATCCTGGCTGT
GATGGCATATGACCGCTATGTGGCCATCTGCTGCCCCCTCCACTACACCA
CAGCCATGAGCCCTAAGCTCTGTATCTTACTCCTTTCCTTGTGTTGGGTC
CTATCCGTCCTCTATGGCCTCATACACACCCTCCTCATGACCAGAGTGAC
CTTCTGTGGGTCACGAAAAATCCACTACATCTTCTGTGAGATGTATGTAT
TGCTGAGGATGGCATGTTCCAACATTCAGATTAATCACACAGTGCTGATT
GCCACAGGCTGCTTCATCTTCCTCATTCCCTTTGGATTCGTGATCATTTC
CTATGTGCTGATTATCAGAGCCATCCTCAGAATACCCTCAGTCTCTAAGA
AATACAAAGCCTTCTCCACCTGTGCCTCCCATTTGGGTGCAGTCTCCCTC
TTCTATGGGACACTTTGTATGGTATACCTAAAGCCCCTCCATACCTACTC
TGTGAAGGACTCAGTAGCCACAGTGATGTATGCTGTGGTGACACCCATGA
TGAATCCCTTCATCTACAGCCTGAGGAACAAGGACATGCATGGGGCTCTG
GGAAGACTCCTAGATAAACACTTTAAGAGGCTGACATGAGGGCAATTTGG
AAAGACAGCATTAAAGTGGAGACTAGGAATATCCTTCACCCTATGTAAGG
GATTGTCCTGTGTGTTATACAGCAGTGATTGGGACATGGCTCCAGCTCAG
AGACAGCATATAGATATGTGGTGATAAAAAAGACATATTTGTAACCTGGT
GTCCCCCAGGTCTCATCAGCCTTGGCCGTAAATAAGGTC
```

PCR primers 1
PCR primers 2
PCR primers 3

Figure S2. RT-PCR analysis of the genes *ACTB, GAPDH, PRM1,* and *HMGB4* from a subset of human sperm samples.

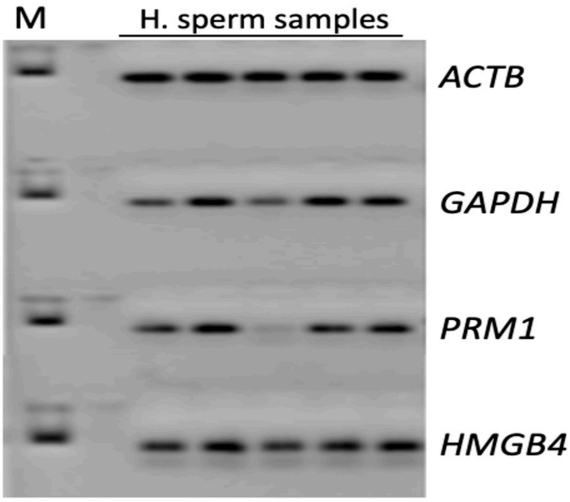

S3 ARRIVE document

<u>The ARRIVE Essential 1.0</u> These items are the basic minimum to include in a manuscript. Without this information, readers and reviewers cannot assess the reliability of the findings.
Item Recommendation
Section/line number, or reason for not reporting

1 Study design
For each experiment, provide brief details of study design including: a. The groups being compared, including control groups. If no control group has been used, the rationale should be stated. b. The experimental unit (e.g. a single animal, litter, or cage of animals).
The objectives were not to compare two groups of samples but to develop a reliable and applicable extraction method of the RNAs present in sperm cells of human and other mammals in order to be able to perform experiments on their role and ultimately determine the implication of these RNAs in male sterility. As detailed in the results section the method we developed is able to extract larger quantity of RNAs and more importantly undegraded RNAs.

2 Sample size
a. Specify the exact number of experimental units allocated to each group, and the total number in each experiment. Also indicate the total number of animals used.
b. Explain how the sample size was decided. Provide details of any a priori sample size calculation, if done

Not really applicable. One can just indicate that we analyzed the content of RNAs of 20 humans, 2 stallions, 2 bulls and 2 dogs of sperm ejaculates.

3 Inclusion and exclusion criteria

a. Describe any criteria used for including and excluding animals (or experimental units) during the experiment, and data points during the analysis. Specify if these criteria were established a priori. If no criteria were set, state this explicitly.

   All ejaculates were obtained from healthy human or animals

b. For each experimental group, report any animals, experimental units or data points not included in the analysis and explain why. If there were no exclusions, state so. c. For each analysis, report the exact value of n in each experimental group

This item is not relevant.

4 Randomisation

a. State whether randomisation was used to allocate experimental units to control and treatment groups. If done, provide the method used to generate the randomisation sequence

This item is not relevant.

b. Describe the strategy used to minimize potential confounders such as the order of treatments and measurements, or animal/cage location. If confounders were not controlled, state this explicitly.

This item is not relevant.

5 Blinding Describe who was aware of the group allocation at the different stages of the experiment (during the allocation, the conduct of the experiment, the outcome assessment, and the data analysis).
This item is not relevant.

6 Outcome measures

a. Clearly define all outcome measures assessed (e.g. cell death, molecular markers, or behavioral changes).

This item is not relevant.

b. For hypothesis-testing studies, specify the primary outcome measure, i.e. the outcome measure that was used to determine the sample size.

All samples were from healthy human or animals.
We considered that with 20 human samples the final results would not be altered if one of them presented a hidden defect. Concerning the animals 2 of each species were the maximum we could get.

7 Statistical methods

a. Provide details of the statistical methods used for each analysis, including software used.

As explained above in item 1, the purpose of the study was to develop an easy and reliable method to extract the RNA content of matured sperm cells from human and other mammals in order to be able to conduct experiments in view of determining their role upon egg fecundation and early embryo development.
In this context, we developed an original method and compared our results with those of methods reported in the literature. To assess the relevance of our method, we compared the RNA yields obtained with these two methods, ours and the classical one.
The yield obtained with the 20 human samples was reported in Table 1a of the submitted article and in Table 1b is the yield obtained with all the samples.
The data obtained from the two extraction methods were statistically analyzed using RStudio to compare the RNA yields and sample quality. Comparisons were made using an unpaired Student's t-test ($p \leq 0.05$).
The analysis, performed using the Welch's t-test, revealed highly significant p-values: $p = 2 \times 10^{-14}$ for humans and $p = 2.22 \times 10^{-16}$ for all mammals (humans, bulls, stallions, and dogs).

b. Describe any methods used to assess whether the data met the assumptions of the statistical approach, and what was done if the assumptions were not met.

As explained above our combined method is superior in term of yield and meet our expectation.

8 Experimental animals

a. Provide species-appropriate details of the animals used, including species, strain and substrain, sex, age or developmental stage, and, if relevant, weight.

This item is not relevant.

b. Provide further relevant information on the provenance of animals, health/immune status, genetic modification status, genotype, and any previous procedures.

As far as one can say all samples were healthy human and animals.

9 Experimental procedures
For each experimental group, including controls, describe the procedures in enough detail to allow others to replicate them, including:
a. What was done, how it was done and what was used.
b. When and how often.
c. Where (including detail of any acclimatisation periods).
d. Why (provide rationale for procedures).
These items are not relevant.

10 Results
For each experiment conducted, including independent replications, report:
a. Summary/descriptive statistics for each experimental group, with a measure of variability where applicable (e.g. mean and SD, or median and range).
b. If applicable, the effect size with a confidence interval.
These items are not applicable.

The ARRIVE guidelines 2.0: author checklist NOTE: Please save this file locally before filling in the table, DO NOT work on the file within your internet browser as changes will not be saved. Adobe Acrobat Reader (available free here) is recommended for completion. The Recommended Set
These items complement the Essential 10 and add important context to the study. Reporting the items in both sets represents best practice. Item Recommendation Section/line number, or reason for not reporting

11 Abstract Provide an accurate summary of the research objectives, animal species, strain and sex, key methods, principal findings, and study conclusions.
As already explained in Study design 1, the aim of this work was to develop a reliable RNA extraction method applicable to different mammalian species. To this end, we compared the efficiency of the extraction of RNAs from human and other mammalian spermatozoa (dog, stallion, and bull) using two distinct approaches: a standard protocol based on a NucleoSpin RNA II kit (Macherey-Nagel) and an optimized protocol that included the addition of DTT (1,4-dithiothreitol) to the RA1 lysis buffer, along with an additional pretreatment step using the TRIzol reagent. Moreover, to ensure the absence of somatic cell contamination, all the semen samples were first purified using a density gradient.

12 Background
a. Include sufficient scientific background to understand the rationale and context for the study, and explain the experimental approach.
b. Explain how the animal species and model used address the scientific objectives and, where appropriate, the relevance to human biology.

Numerous studies have clearly demonstrated that sperm RNAs are not mere transcriptional remnants of spermatogenesis; rather, they play essential roles in male fertility, fertilization, early embryonic development, and epigenetic inheritance Therefore we are convinced that one need a reliable method to extract the largest possible amount of non-degraded RNAs in view of performing experimental studies regarding their exact rules.

13 Objectives Clearly describe the research question, research objectives and, where appropriate, specific hypotheses being tested.
Already described above.

14 Ethical statement Provide the name of the ethical review committee or equivalent that has approved the use of animals in this study, and any relevant licence or protocol numbers (if applicable). If ethical approval was not sought or granted, provide a justification. Housing and husbandry

Samples were provided by the GERMETHEQUE Biobank, dedicated to human fertility. Semen human samples (n=20) were obtained from men consulting the CECOS laboratory (Center for the Study and Conservation of Human Eggs and Sperm) in Rennes. All samples had normal spermograms. Patients were informed about the nature of the study and provided informed consent for the residual portion of their samples to be used after spermogram and spermocytogram analyses. Ethical approval (CP-GM n°20151117) was obtained for this study. Additionally, semen samples were collected from 2 bulls, 2 stallions and 2 dogs at Nantes Veterinary School's reproduction service.

15 Provide details of housing and husbandry conditions, including any environmental enrichment. Animal care and monitoring
Not applicable

16 a. Describe any interventions or steps taken in the experimental protocols to reduce pain, suffering and distress.
b. Report any expected or unexpected adverse events.
c. Describe the humane endpoints established for the study, the signs that were monitored and the frequency of monitoring. If the study did not have humane endpoints, state this. Interpretation/ scientific implications

Not relevant

17 a. Interpret the results, taking into account the study objectives and hypotheses, current theory and other relevant studies in the literature
As already detailed above our objectives were attained
b. Comment on the study limitations including potential sources of bias, limitations of the animal model, and imprecision associated with the results.
Not relevant

18 Generalisability/ translation Comment on whether, and how, the findings of this study are likely to generalise to other species or experimental conditions, including any relevance to human biology (where appropriate)
As already said the goal of the study was to develop a reliable method to extract the RNAs from matured sperm cell of human as well as some relevant mammals r for studying the causes of sterility in presence of normal spermogram. As such our results are fully generalizable.

19 Protocol registration Provide a statement indicating whether a protocol (including the research question, key design features, and analysis plan) was prepared before the study, and if and where this protocol was registered.
The protocol was not registered. It was discussed in depth between El oulidi Mounia, Azzouzi Naoual, Ravel Celia and myself before we started to collect the samples.

20 Data access Provide a statement describing if and where study data are available.
The data will be fully available upon publication.

21 Declaration of interests
a. Declare any potential conflicts of interest, including financial and non-financial. If none exist, this should be stated.

There is no conflict of interest for any of us
b List all funding sources (including grant identifier) and the role of the funder(s) in the design, analysis and reporting of the study.
We did not get any additional funding to support this work which was mainly done using the regular support of the laboratory which depends of the University and CNRS, as well as from all Universities of Morocco.
